# Vulvar Metastasis From Colorectal Adenocarcinoma: A Report of a Rare Case and Review of Diagnostic and Therapeutic Approaches

**DOI:** 10.7759/cureus.108140

**Published:** 2026-05-02

**Authors:** Kaouthar Khater, Hanan Bailal, Fadoua Jebrouni, Asmae Bali, Hind Chibani, Ouissam Al Jarroudi, Sami Aziz Brahmi, Said Afqir

**Affiliations:** 1 Medical Oncology, Mohammed VI University Hospital, Faculty of Medicine and Pharmacy of Oujda, Mohammed First University of Oujda, Oujda, MAR

**Keywords:** cdx2, immunohistochemistry, metastatic colorectal cancer, rectal adenocarcinoma, vulvar metastasis

## Abstract

Vulvar metastasis from colorectal adenocarcinoma is extremely rare, with few cases reported in the literature. We present the case of a 49-year-old woman with a history of locally advanced, moderately to well-differentiated invasive adenocarcinoma of the distal rectum treated with neoadjuvant chemotherapy and abdominoperineal resection, including partial posterior vaginal wall resection. One year later, she developed a vulvar lesion associated with metrorrhagia. Pelvic magnetic resonance imaging showed an isolated vulvar lesion without pelvic recurrence. Histopathology and immunohistochemistry confirmed metastatic colorectal adenocarcinoma. A multidisciplinary decision led to systemic chemotherapy with irinotecan, leucovorin, and 5-fluorouracil plus bevacizumab.

This case highlights the importance of integrating clinical evaluation, histopathology, immunohistochemistry, and imaging in diagnosing rare metastatic sites and emphasizes the role of multidisciplinary board decisions in guiding systemic therapy and optimizing patient management.

## Introduction

Rectal cancer represents a significant proportion of colorectal malignancies, accounting for approximately 28% of cases, second only to proximal colon cancers at 42% [1]. Overall, colorectal cancer is a major global public health concern and ranks among the most common cancers worldwide, with an estimated lifetime risk of 4.7%-5% [2]. Despite advances in early detection and multimodal therapy, including neoadjuvant chemoradiotherapy and surgical techniques, rectal cancer continues to have a significant risk of recurrence and metastasis [3].

The most frequent sites of colorectal metastases are the liver, lungs, and peritoneum, reflecting the venous drainage and lymphatic pathways of the colorectal region [4]. Involvement of the female lower genital tract, including the vulva and vagina, is rare, with most evidence derived from case reports and small case series [5]. Primary vulvar malignancies are uncommon and are predominantly squamous cell carcinomas. In contrast, primary vulvar adenocarcinomas are rare and most often arise from the Bartholin glands or are associated with extramammary Paget’s disease. A potential risk of local recurrence should also be considered in proximity to surgical resection margins, particularly involving the posterior vaginal wall adjacent to the vulvar region [6].

Given the rarity of vulvar metastases from colorectal adenocarcinoma, awareness of this entity is essential to avoid misdiagnosis, which can lead to inappropriate local therapies, delayed systemic treatment, or incorrect staging. This case may increase clinicians’ awareness of this rare entity and support earlier recognition, leading to prompt biopsy, accurate diagnosis, and appropriate multidisciplinary management [7].

## Case presentation

A 49-year-old woman with a history of diabetes was diagnosed with locally advanced, moderately to well-differentiated invasive adenocarcinoma of the distal rectum, staged as cT3N1. Following a multidisciplinary tumor board discussion, she received total neoadjuvant chemotherapy according to a total neoadjuvant therapy (TNT) strategy, using a regimen consisting of oxaliplatin (130 mg/m² administered intravenously on day 1) and capecitabine (1000 mg/m² taken orally twice daily from day 1 to day 14, every three weeks). The patient completed six cycles of chemotherapy, with the final cycle administered in March 2022.

She was subsequently referred to the Surgical Department for abdominoperineal resection with pseudo-continent colostomy. However, surgery was initially delayed due to the patient’s refusal, and she was temporarily lost to follow-up. The procedure was eventually performed in October 2022 and consisted of an abdominoperineal resection with partial resection of the lower third of the posterior vaginal wall.

**Figure 1:**
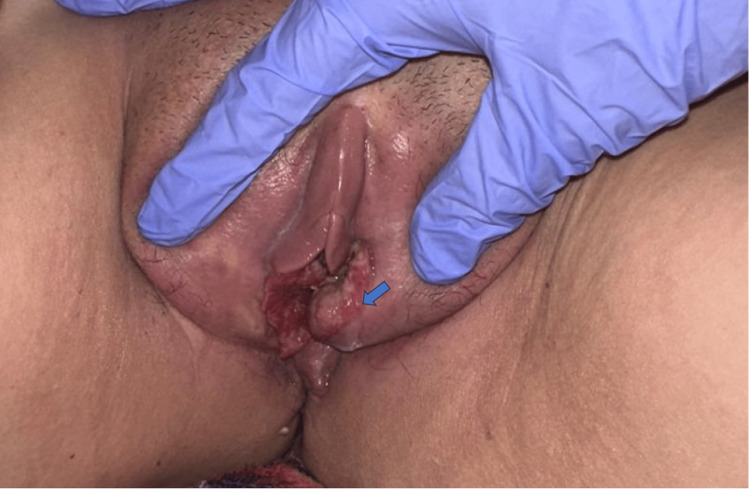
Gynecological examination showing an ulcerated and infiltrative vulvar mass (arrow).

**Figure 2:**
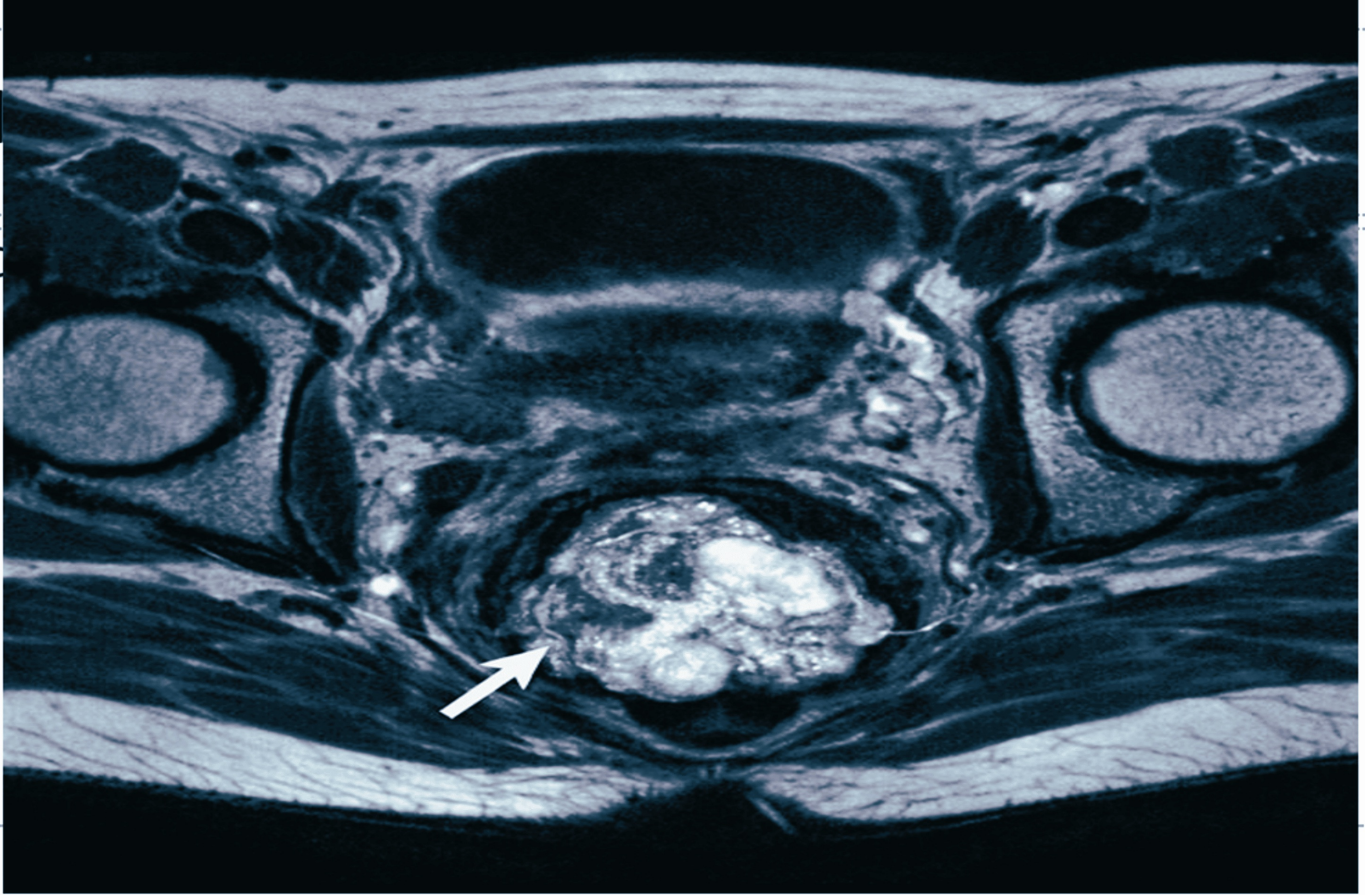
MRI pelvic axial T2-weighted image. MRI pelvic axial T2-weighted image demonstrating a lobulated vulvar lesion with heterogeneous signal intensity (arrow), measuring approximately 57 × 54 mm with a craniocaudal extension of about 45 mm. No extension to the vagina, urethra, or pelvic lymph nodes was observed, supporting the diagnosis of an isolated vulvar metastatic lesion. MRI: magnetic resonance imaging

One year later, during follow-up, the patient presented with metrorrhagia associated with a vulvar lesion (Figure 1). Pelvic magnetic resonance imaging (MRI) was performed for further lesion characterization. On T2-weighted images, the mass appeared heterogeneously hyperintense with a lobulated contour in the vulvar region. Following contrast administration, it demonstrated heterogeneous enhancement. No extension to adjacent structures, including the vagina, urethra, or surrounding pelvic tissues, was observed, and no pelvic lymphadenopathy was identified (Figure 2).

**Figure 3:**
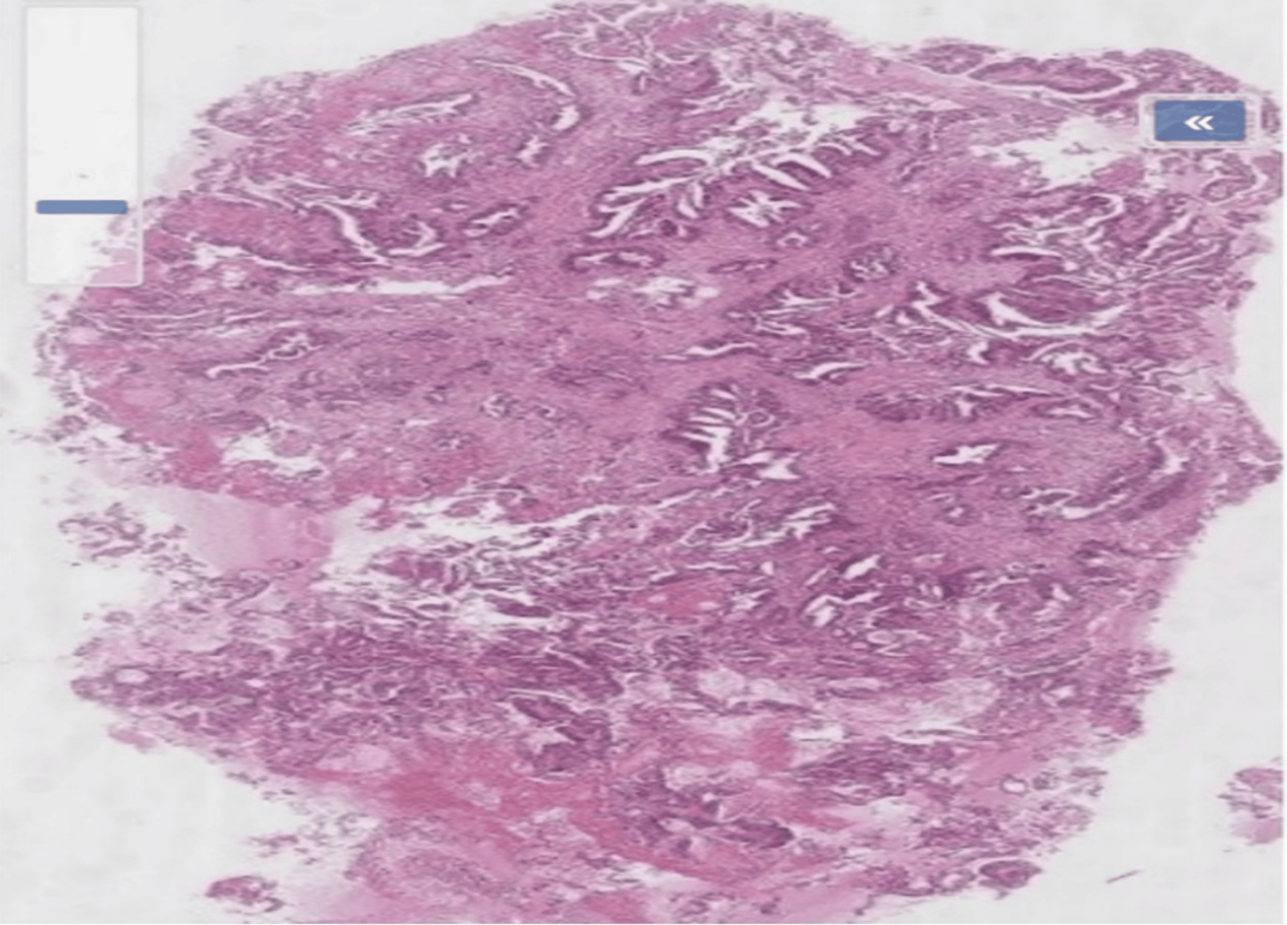
Histological section of the vulvar lesion stained with hematoxylin and eosin (H&E) at low magnification. Histological section of the vulvar lesion stained with H&E at low magnification showing a moderately to well-differentiated adenocarcinoma with glandular and papillary architecture infiltrating the vulvar stroma. The tumor exhibits irregular gland formation and nuclear atypia consistent with metastatic colorectal adenocarcinoma (H&E stain, ×40).

**Figure 4:**
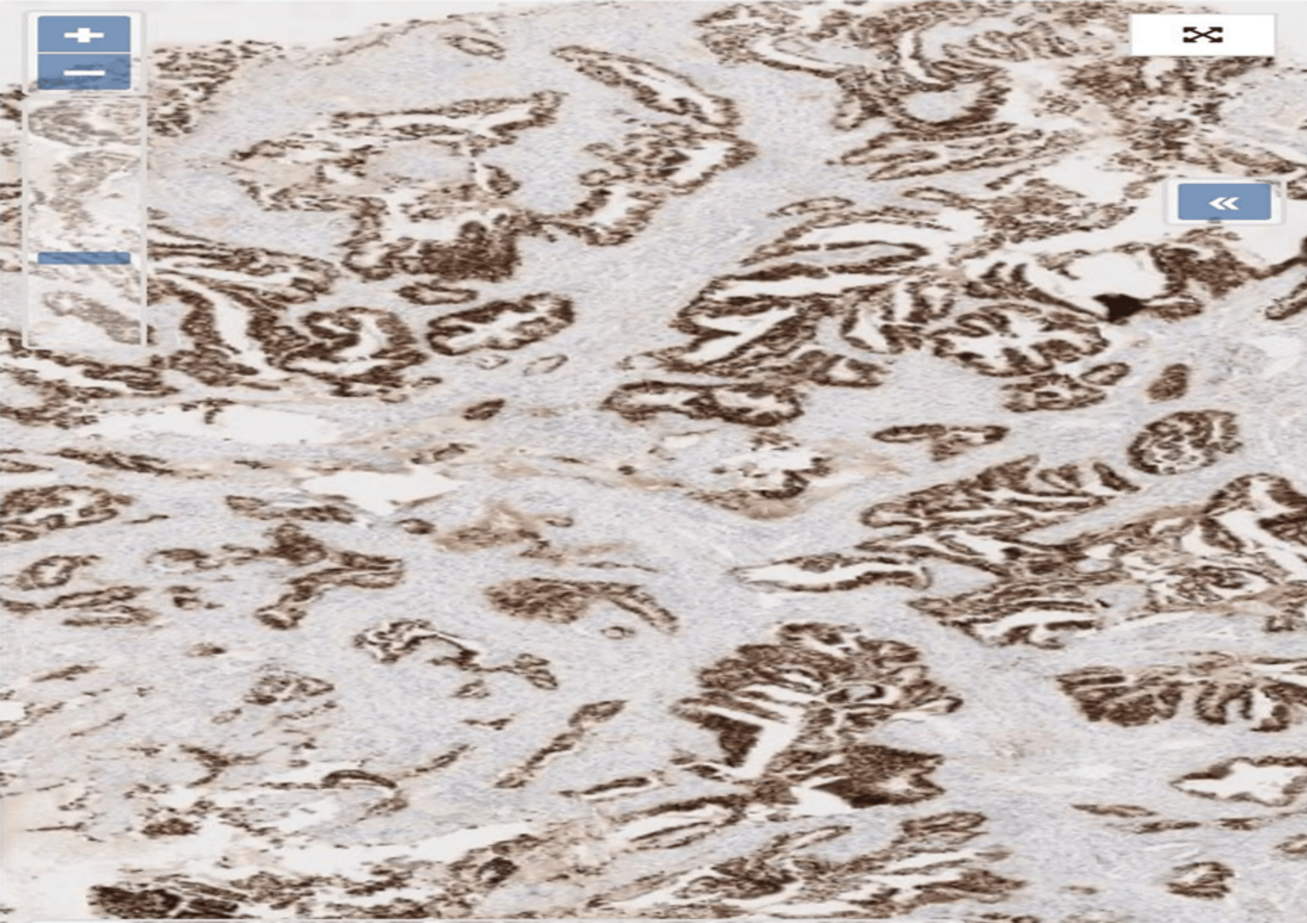
Immunohistochemical staining at high magnification. Immunohistochemical staining at high magnification demonstrating strong, diffuse nuclear positivity for CDX2 in tumor cells arranged in glandular and papillary structures, consistent with metastatic colorectal adenocarcinoma to the vulva (CDX2 stain, ×40). CDX2: caudal-related homeobox gene 2

A vulvar biopsy was subsequently performed. Histopathological examination revealed infiltration by a moderately to well-differentiated adenocarcinoma. Immunohistochemical analysis demonstrated strong nuclear positivity for caudal-related homeobox gene 2 (CDX2), confirming a vulvar metastasis of colorectal origin (Figures 3-4).

Following multidisciplinary discussion, the patient was started on first-line systemic chemotherapy consisting of irinotecan, leucovorin, and 5-fluorouracil (FOLFIRI), combined with bevacizumab, with good tolerance and clinical stability at the most recent follow-up.

## Discussion

Rectal cancer is one of the most common malignancies worldwide, accounting for approximately 28% of colorectal cancers, behind proximal colon cancers (42%) [1]. It represents a significant public health concern and is generally included in epidemiological studies on colorectal cancer, which ranks as the third most frequent cancer in men and the second in women, with a lifetime risk of 4.7%-5% [5]. Rectal cancer is distinguished by its anatomical location and its typical metastatic pattern, most frequently to the liver, lungs, and peritoneum. Metastatic involvement of the lower female genital tract, particularly the vulva and vagina, is extremely uncommon and accounts for less than 1% of metastatic manifestations of colorectal cancer [6,8].

Primary vulvar malignancies are relatively uncommon and represent a small proportion of gynecologic cancers. Among them, squamous cell carcinoma is by far the most frequent histological subtype, accounting for approximately 86%-90% of all vulvar cancers. These tumors are often associated with either human papillomavirus (HPV) infection or chronic inflammatory conditions, such as lichen sclerosus [9]. In contrast, primary vulvar adenocarcinomas are rare, representing about 5%-8% of tumors and typically arising from Bartholin glands or in association with extramammary Paget’s disease [10,11].

The clinical manifestation of vulvar or vaginal metastases from colorectal cancer is often nonspecific, including a vulvar mass, metrorrhagia, discharge, or pelvic discomfort [8]. In the present case, the patient developed a vulvar lesion with metrorrhagia one year after surgery, which led to clinical examination and biopsy. Thus, careful clinical evaluation is essential, as any new vulvar lesion in patients with a history of rectal cancer should raise suspicion for metastatic disease [12].

Given these nonspecific clinical presentations, understanding the potential metastatic pathways becomes essential for accurate diagnosis. Vulvar metastases most commonly occur through lymphatic or hematogenous dissemination. Tumor cells may spread through rectal and mesorectal lymph nodes, with possible retrograde dissemination to vulvar lymphatics. Hematogenous spread via the pelvic venous system to the vulvar venous plexus represents another potential pathway, allowing tumor cells to reach the vulvar region. Direct tumor extension appears unlikely in this case, as the vulvar lesion developed one year after surgery without evidence of pelvic recurrence, suggesting hematogenous or lymphatic dissemination as the most probable mechanism. Recognition of these pathways provides a useful framework for anticipating metastatic patterns and guiding follow-up in patients with rectal adenocarcinoma [8,13,14].

Radiological evaluation plays a pivotal role in evaluating vulvar lesions and excluding local recurrence. Pelvic MRI provides a comprehensive assessment of lesion size, morphology, local extension, and involvement of adjacent structures [9,15]. In this patient, pelvic MRI demonstrated a solitary vulvar mass without extension to the vagina, urethra, or pelvic lymph nodes, supporting distant metastatic spread rather than direct invasion. On T2-weighted sequences, the lesion showed hyperintense signals characteristic of adenocarcinoma, and contrast-enhanced sequences revealed heterogeneous enhancement. These imaging findings complemented histopathology and immunohistochemistry, guiding biopsy and timely initiation of systemic therapy [9,16].

The histopathological distinction between metastatic colorectal adenocarcinoma and primary vulvar adenocarcinoma is often challenging due to overlapping morphological features. Although metastatic lesions may exhibit glandular structures, mucin production, and nuclear stratification suggestive of colorectal origin, these findings are not specific [17,18]. Immunohistochemical analysis, therefore, plays a crucial role in establishing the diagnosis. In the present case, the tumor demonstrated a moderately to well-differentiated adenocarcinoma infiltrating the stroma, with strong nuclear expression of CDX2, supporting an intestinal origin [19]. The combined use of cytokeratin 7 (CK7) and cytokeratin 20 (CK20) further contributes to differentiating metastatic colorectal disease from primary vulvar tumors, guiding appropriate clinical management [6,20,21]. 

Systemic therapy remains the cornerstone of management for metastatic colorectal cancer, particularly when the disease occurs at uncommon extraintestinal sites such as the vulva. Unlike primary vulvar malignancies, which are generally treated with local approaches including surgery and radiotherapy, metastatic colorectal disease requires a systemic therapeutic strategy [11,22]. In this case, treatment decisions were guided by a multidisciplinary tumor board, taking into account prior therapies, clinical status, and disease characteristics. Given the history of oxaliplatin-based chemotherapy and surgery, the vulvar lesion was considered a manifestation of systemic recurrence. First-line systemic therapy was initiated in accordance with current guidelines, consisting of FOLFIRI combined with bevacizumab, with the aim of enhancing therapeutic efficacy, improving progression-free survival, and inhibiting tumor angiogenesis [23,24].

For a significant period, vaginal metastasis from colorectal cancer has been associated with a poor prognosis. However, recent evidence suggests that a more aggressive diagnostic and therapeutic approach may improve outcomes and potentially contribute to prolonged survival in selected patients [13].

## Conclusions

Vulvar metastasis from colorectal adenocarcinoma is an exceptionally rare and often unexpected manifestation of metastatic disease. This case underscores the need for high clinical suspicion when new vulvar lesions appear in patients with a history of colorectal cancer. Accurate diagnosis requires integration of clinical history, imaging, histopathology, and immunohistochemistry. Early recognition is essential, as management differs from primary vulvar malignancies, with systemic therapy forming the cornerstone. Reporting such rare cases enhances understanding of atypical metastatic pathways and supports timely, appropriate patient management.
